# Mesenteric Mass Causing Bowel Obstruction in Waldenström Macroglobulinemia

**DOI:** 10.7759/cureus.88091

**Published:** 2025-07-16

**Authors:** Eduarda Magalhães, Paulo Sousa, Jose Pedro Pinto, Fernando Manso, Ana Cristina Ribeiro

**Affiliations:** 1 General Surgery, Unidade Local de Saúde de Braga, Braga, PRT; 2 Bariatric Surgery, Unidade Local de Saúde de Braga, Braga, PRT

**Keywords:** abdominal mass, intestinal obstruction, lymphoplasmacytic lymphoma, mesenteric involvement, waldenström macroglobulinemia

## Abstract

Waldenström macroglobulinemia (WM) is a rare, indolent lymphoplasmacytic lymphoma characterized by bone marrow infiltration and monoclonal immunoglobulin M (IgM) production. While WM is usually characterized by slow clinical progression, the occurrence of abdominal masses leading to intestinal obstruction is an exceptional manifestation.

We report the case of a 62-year-old man referred for evaluation of a pulmonary lesion, with positron emission tomography-computed tomography (PET-CT) findings suggestive of a systemic lymphoproliferative process involving the abdomen and mesentery. Bone marrow immunophenotyping confirmed WM, and histological examination of an abdominal mass revealed a low-grade B-cell non-Hodgkin lymphoma. Initial treatment with bendamustine and rituximab was started. However, the patient developed recurrent abdominal cramps, weight loss, and progressive signs of bowel obstruction, culminating in a laparoscopic segmental enterectomy. Histopathology of the resected specimen demonstrated transmural intestinal ischemia and fibrosis, but no residual lymphoma. Postoperatively, the patient had an uneventful recovery with resolution of symptoms. This case highlights a rare and severe gastrointestinal complication of WM requiring surgical intervention despite early chemotherapy. It emphasizes the importance of a multidisciplinary approach when managing atypical presentations of hematologic malignancies.

## Introduction

Waldenström macroglobulinemia (WM) is a rare, indolent B-cell lymphoproliferative disorder, specifically a lymphoplasmacytic lymphoma, characterized by bone marrow infiltration by clonal lymphoplasmacytic cells and the production of monoclonal immunoglobulin M (IgM) [1,2]. Its incidence is low, estimated at approximately three to four cases per million persons per year [3]. The pathogenesis of WM is frequently associated with the somatic L265P mutation in the MYD88 gene, present in over 90% of patients, which activates pro-survival signaling pathways essential for tumor cell growth [4]. Typical clinical manifestations can arise from bone marrow infiltration (leading to cytopenias such as anemia), the physicochemical properties of the monoclonal IgM (e.g., hyperviscosity syndrome, cryoglobulinemia, peripheral neuropathy), or constitutional "B" symptoms [1,5].

While extranodal involvement can occur in sites such as the lymph nodes, spleen, and liver, gastrointestinal (GI) tract involvement in WM is considered infrequent, reported in a small percentage of cases [2,5]. Patients may present with a spectrum of GI symptoms; the most commonly seen symptom is diarrhea. Other symptoms include abdominal pain, malabsorption, gastrointestinal bleeding, and weight loss [6].

Few cases in the literature describe gastrointestinal involvement in WM with histological confirmation, and even fewer report mass-forming lesions. The majority of GI manifestations are related to mucosal infiltration, amyloidosis, or immunoglobulin deposition. Heenan et al. described a case of partial small bowel obstruction due to jejunal involvement by WM [7], and isolated reports exist of protein-losing enteropathy and colitis secondary to lymphoplasmacytic infiltration [8]. However, to our knowledge, no previous reports describe a bulky mesenteric mass leading to mechanical subocclusion requiring surgical resection in the context of WM.

We report a case of WM presenting with an extensive mesenteric mass, leading to recurrent subocclusive episodes and ultimately requiring surgical resection - an exceptionally rare manifestation not previously described in the literature.

## Case presentation

A 62-year-old male patient, with a medical history of hypertension, dyslipidemia, and previous gastric ulcers, was referred to the Hematology clinic in August 2024 following the incidental identification of a pulmonary lesion during investigation of chronic cough. He had no known allergies, was a former smoker, and had no relevant family history of hematologic malignancy. In the preceding months, he reported progressive fatigue and unintentional weight loss of approximately 6 kg, without associated fever, night sweats, or gastrointestinal complaints. Physical examination was unremarkable, and he was not on immunosuppressive therapy.

Initial laboratory investigations revealed normocytic anemia, elevated erythrocyte sedimentation rate (ESR), increased total serum protein with a reversed albumin-globulin ratio, and an IgM monoclonal spike on serum protein electrophoresis (Table 1). Further evaluation with bone marrow aspirate and flow cytometry identified a small clonal population of B cells and plasmacytoid cells expressing IgM kappa light chains, compatible with lymphoplasmacytic lymphoma/WM. MYD88 mutation testing was performed and returned negative. CXCR4 testing was not performed due to institutional limitations. Nevertheless, the absence of a CXCR4 analysis did not preclude diagnostic or therapeutic decisions, as the overall clinicopathological and immunophenotypic profile remained strongly suggestive of WM.

**Table 1:** Relevant laboratory findings at diagnosis IgM: immunoglobulin M; LDH: lactate dehydrogenase

Parameter	Patient Value	Reference Range
Hemoglobin	10.4 g/dL	13.5 – 17.5 g/dL
White Blood Cell Count	6.2 × 10⁹/L	4.0 – 11.0 × 10⁹/L
Platelet Count	180 × 10⁹/L	150 – 400 × 10⁹/L
ESR	72 mm/h	< 20 mm/h
Total Protein	9.1 g/dL	6.4 – 8.3 g/dL
Albumin	3.1 g/dL	3.5 – 5.0 g/dL
Globulin	6.0 g/dL	2.0 – 3.5 g/dL
Albumin/Globulin Ratio	0.52	~1.0 – 2.0
IgM	4,200 mg/dL	40 – 230 mg/dL
Serum Protein Electrophoresis	Monoclonal IgM spike	—
Immunofixation	Monoclonal IgM kappa	—
Beta-2 microglobulin	4.8 mg/L	1.0 – 2.4 mg/L
LDH	390 U/L	135 – 225 U/L
Creatinine	1.0 mg/dL	0.7 – 1.3 mg/dL
Calcium	9.0 mg/dL	8.6 – 10.2 mg/dL
MYD88 Mutation	Negative	—

**Figure 1:**
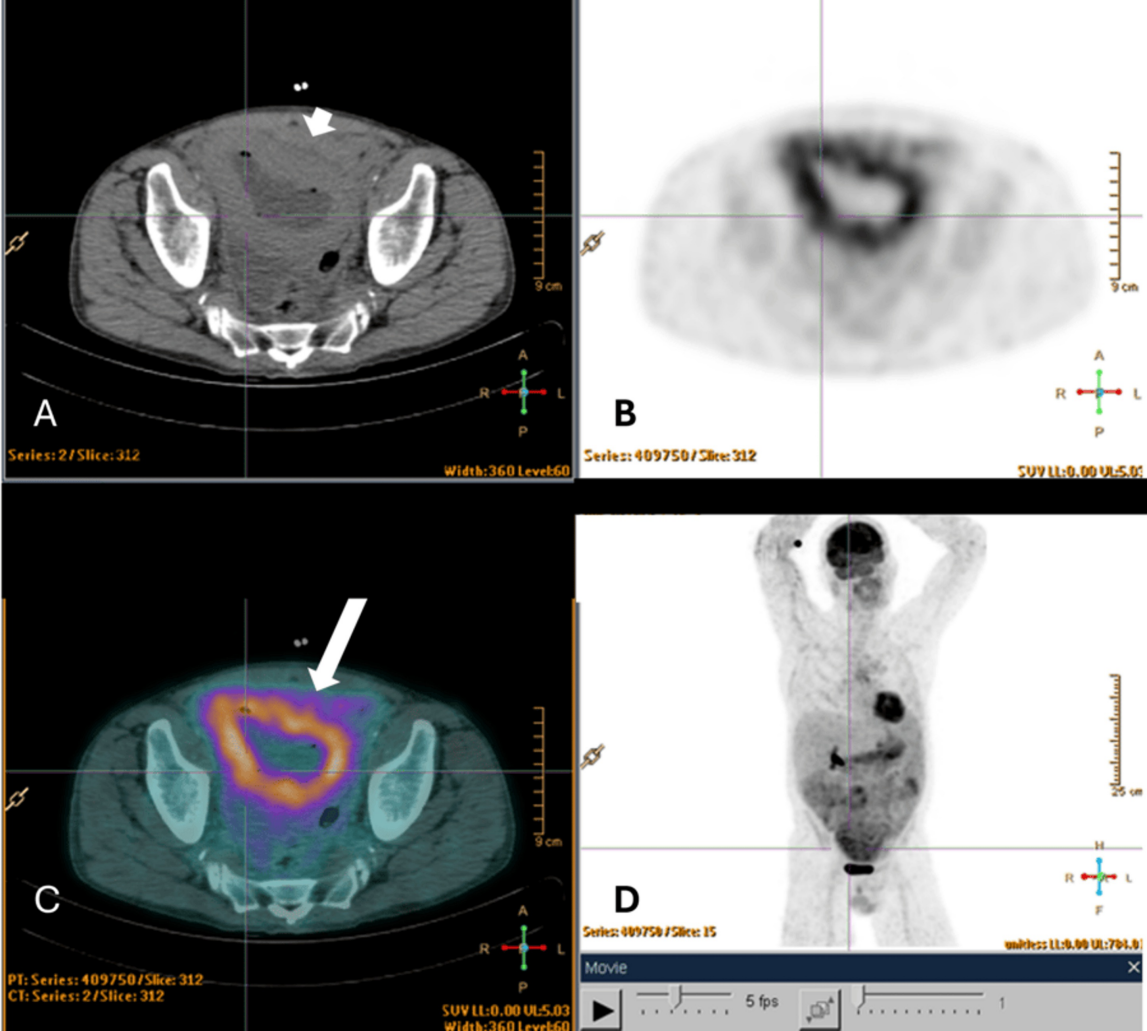
Whole-body PET-CT scan (fourth week of August) A. Axial low-dose CT: Low-dose axial CT showing segmental circumferential wall thickening of pelvic small bowel loops (thin white arrow), suggesting inflammatory or lymphomatous infiltration. B. Axial PET: PET image demonstrating increased 18F-FDG uptake in the same segment of small bowel, without clear anatomical boundaries. C. Axial PET-CT fusion: Fused PET-CT image confirming concordant FDG uptake in the thickened intestinal loops (thick white arrow), with an SUVmax of 5.03. D. Whole-body MIP image: Diffuse and heterogeneous increased uptake of the radiotracer is observed throughout the abdominal cavity. Due to the limitations of the current low-dose, non-contrast CT, anatomical correlation is challenging. However, extensive peritoneal effusion is identified, along with apparent fat stranding. Areas of more intense uptake are noted in the projection of small bowel loops and within the peritoneal fluid and fat stranding, particularly in the epigastric/left hypochondrium and hypogastric regions. PET-CT: positron emission tomography-computed tomography; FDG: fluorodeoxyglucose; SUVmax: maximum standardized uptake value

A positron emission tomography-computed tomography (PET-CT) scan revealed hypermetabolism with increased 18F-FDG uptake in areas of pulmonary consolidation, mediastino-hilar lymphadenopathy, and diffusely throughout the abdominal cavity. The spleen also exhibited increased heterogeneous uptake, and a mesenteric mass with infiltrative features was identified (Figure 1).

**Figure 2:**
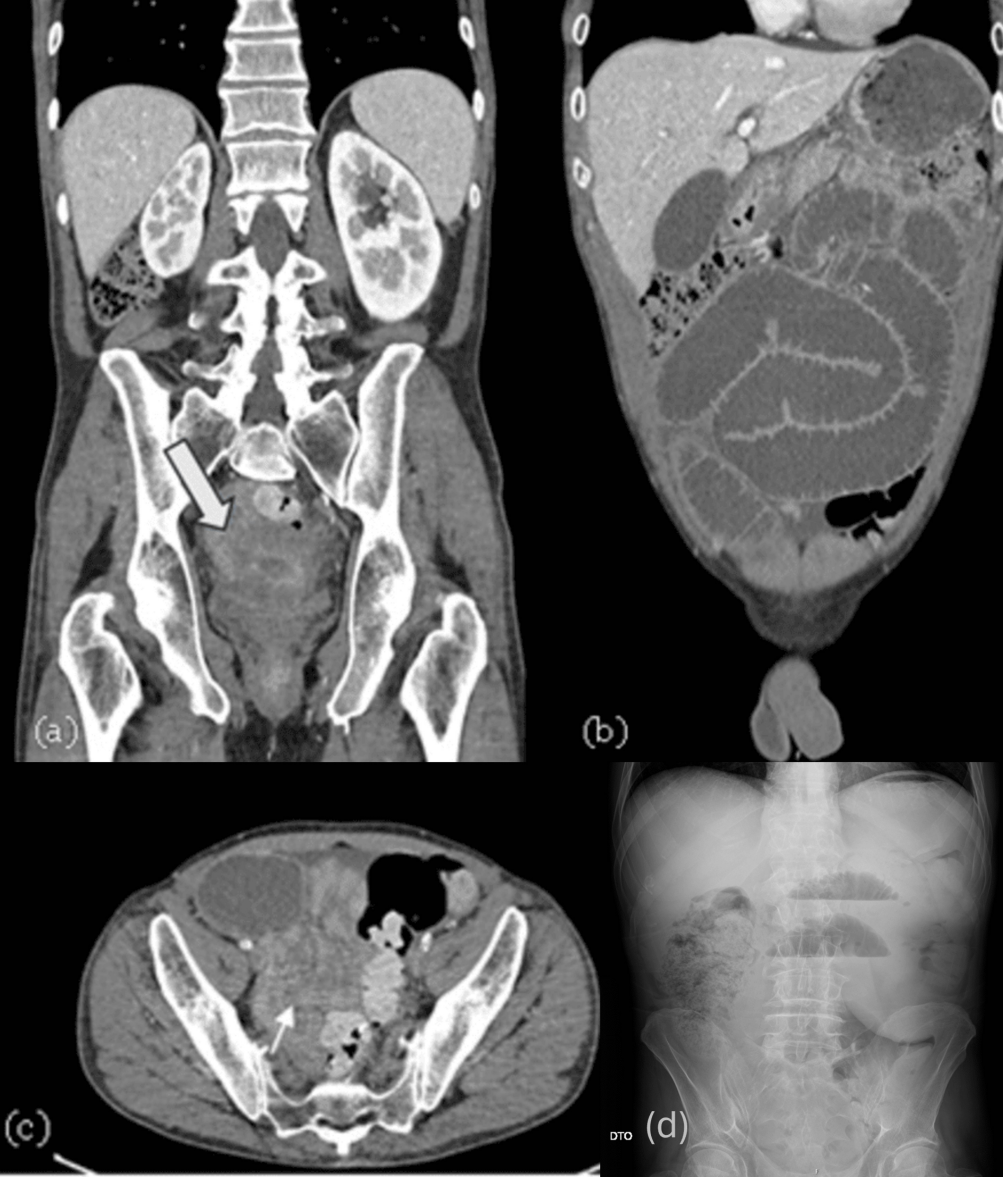
Abdominopelvic CT scan with intravenous contrast (a, b, c) and abdominal radiograph (d), showing a pelvic mass with subocclusive features (a) Coronal reconstruction identifying a soft-tissue pelvic mass (as identified by the arrow) displacing adjacent small bowel loops. (b) Sagittal view illustrating the cranio-caudal extension of the mass and associated proximal small bowel distension. (c) Axial section confirming circumferential displacement and compression of the intestinal lumen by the mesenteric lesion (as identified by the arrow). (d) Supine abdominal radiograph showing multiple dilated small bowel loops with air-fluid levels, predominantly in the central abdomen, consistent with mechanical small bowel obstruction.

The diagnosis of WM was confirmed through a combination of histological, immunophenotypic, and molecular data. Bone marrow aspirate and flow cytometry identified a clonal population of B cells and plasmacytoid cells expressing IgM and kappa light chains. Immunohistochemical analysis of the abdominal mass showed diffuse positivity for CD20 and CD79a, focal CD5 expression, and negativity for Cyclin D1, CD3, and CD4, supporting a diagnosis of low-grade B-cell non-Hodgkin lymphoma (grade 1). MYD88 mutation testing was performed and returned negative. CXCR4 testing was not documented. These findings, together with the clinical and laboratory features, were consistent with WM with extensive abdominal involvement. During a multidisciplinary team consultation at the end of September 2024, first-line chemoimmunotherapy was initiated with the R-Benda regimen, consisting of rituximab 375 mg/m² IV on day 1 and bendamustine 90 mg/m² IV on days 1 and 2, administered with appropriate premedication. This regimen was repeated every 28 days for a planned total of six cycles. In November 2024, the patient began experiencing complaints of abdominal cramps, which subsided with symptomatic treatment. In January 2025, after multiple emergency department visits due to abdominal cramps, absence of bowel movements, and weight loss, and following repeated refusals of surgical intervention, the patient once again presented to the emergency department with progressive abdominal pain, abdominal distension, and clinical signs of intestinal obstruction. An abdominal radiograph and abdominopelvic computed tomography (CT) scan were performed, revealing worsening distension of intestinal loops compared to previous examinations, and the presence of the previously identified pelvic mass (Figure 2).

**Figure 3:**
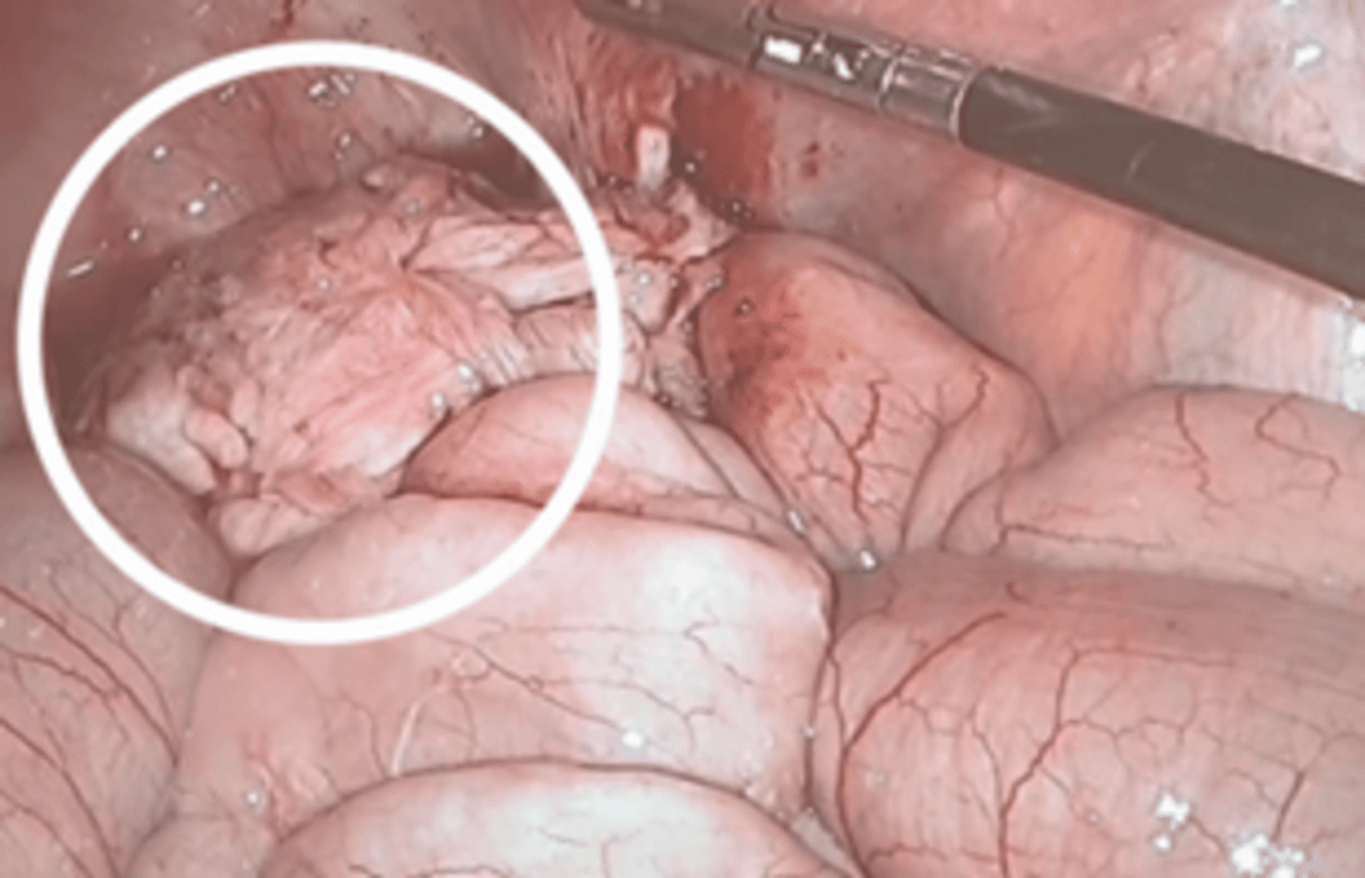
Intraoperative laparoscopic view of the abdominal cavity showing a firm, irregular mesenteric mass (circle) with infiltration of adjacent small bowel loops The mass was the source of recurrent subocclusive symptoms and was surgically resected.

Given the clinical picture of recurrent sub-occlusive episodes and the lack of regression in the size of the mass despite the treatments that were administered (having completed two cycles at that point), the patient underwent a laparoscopic segmental enterectomy in the second week of January 2025 (Figure 3).

**Figure 4:**
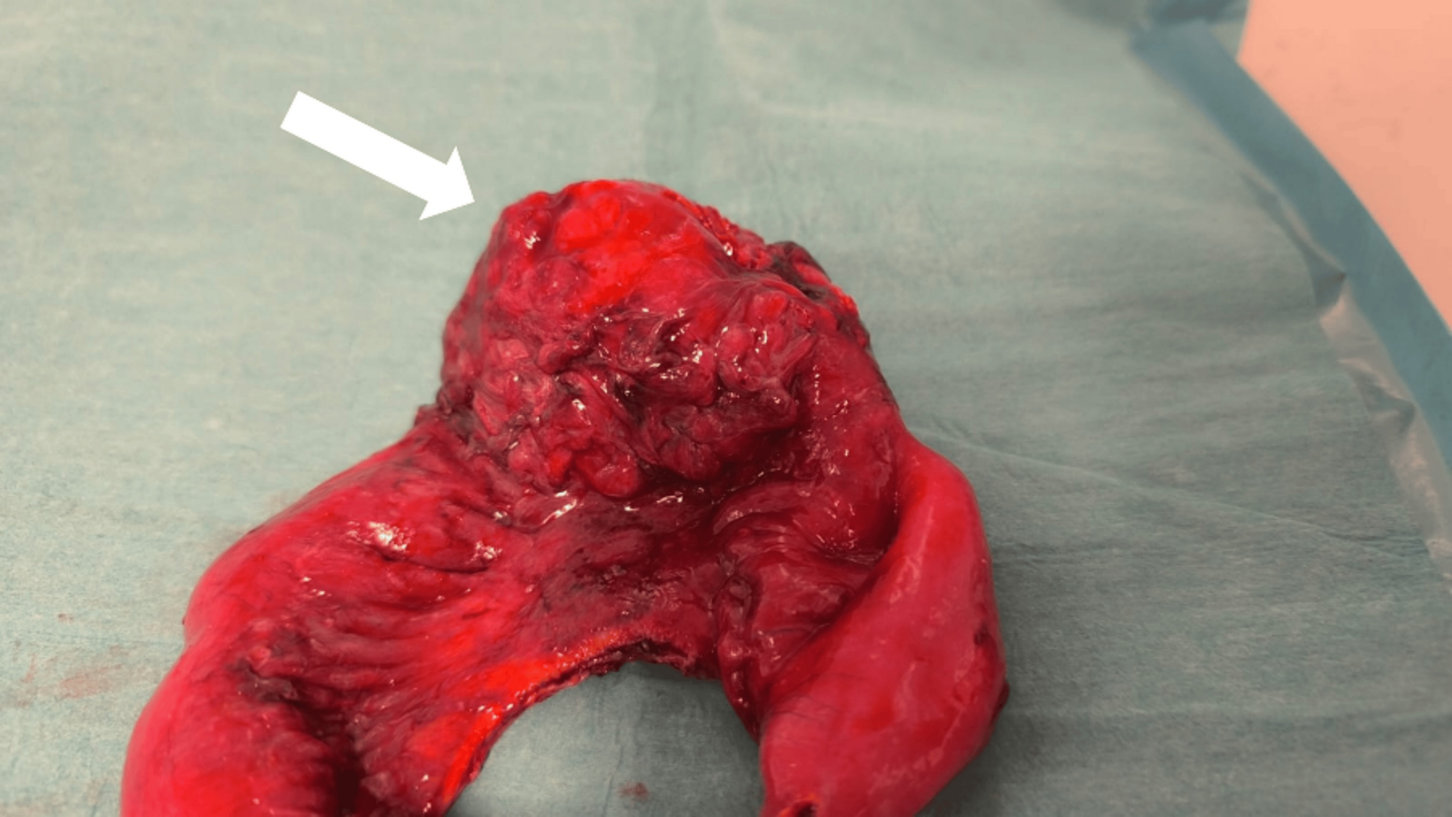
Gross pathological specimen of the resected small bowel showing a fibrotic, irregular mass (arrow) involving the mesentery and bowel wall Despite its macroscopic tumor-like appearance, histopathological analysis revealed transmural ischemia and extensive fibrosis.

The surgical procedure included resection of the mass along with the involved intestinal loop, followed by a side-to-side entero-enteric anastomosis. The gross pathological specimen revealed a fibrotic, irregular mass involving the mesentery and bowel wall, with a macroscopic tumor-like appearance (Figure 4). However, histopathological analysis of the resected tissue demonstrated extensive transmural ischemia and marked fibrosis, with no evidence of residual lymphomatous infiltration. Digital histological images were not available for inclusion in the manuscript, as the pathological examination was conducted in an external reference laboratory not affiliated with our institution.

The patient experienced a favorable postoperative course, with progressive improvement in inflammatory parameters, tolerance to oral intake, and restoration of bowel function. He was discharged on postoperative day six, with dietary guidance and clinical follow-up recommendations. At the time, the patient remained under active clinical surveillance while completing the planned R-Benda chemoimmunotherapy regimen, with no recurrence of obstructive symptoms and stable overall condition. The long-term outcome is not yet established.

## Discussion

WM is an indolent B-cell lymphoma where significant gastrointestinal (GI) mass formation leading to mechanical bowel obstruction is an exceedingly rare event [2,3]. While extranodal involvement in WM is recognized, including instances of GI infiltration [7-12], the literature supporting WM as a direct cause of high-grade mechanical bowel obstruction due to a discrete mass is sparse. Notably, Heenan P.J. et al. described a case of WM with jejunal involvement leading to partial obstruction [7], highlighting that such intestinal complications, though rare, can occur. Our case adds to this limited body of evidence, presenting a patient with a bulky mesenteric mass causing progressive subocclusion, and offers unique insights, particularly regarding the post-chemotherapy histopathological findings.

The typical GI involvement in WM, when described more broadly, usually pertains to diffuse lymphoplasmacytic infiltration of the mucosa and submucosa, or complications like AL amyloidosis or cryoglobulinemic vasculitis affecting the GI tract [3,10]. The formation of a solid, obstructive mesenteric tumor, as seen in our patient and in a distinct manner in the case reported by Heenan et al., represents a more aggressive local manifestation and poses unique diagnostic and therapeutic challenges [7].

The differential diagnosis of an IgM-secreting lymphoproliferative disorder includes marginal zone lymphoma, mantle cell lymphoma, and chronic lymphocytic leukemia. In our case, the immunophenotypic profile - CD20+, CD79a+, focal CD5+, Cyclin D1−, CD3−, CD4− - was not consistent with mantle cell lymphoma (excluded by negative Cyclin D1 and absence of t(11;14)), nor with other small B-cell neoplasms. Furthermore, the detection of a clonal B-cell population expressing IgM and kappa light chains in the bone marrow, along with the presence of a monoclonal IgM spike and compatible clinical features, strongly supported the diagnosis of WM [4].

Although the MYD88 L265P mutation, present in approximately 90-95% of WM cases, was not detected, this does not exclude the diagnosis. As highlighted in current literature, MYD88-negative WM cases do occur and require integration of histologic, immunophenotypic, and clinical data for accurate classification [4]. In this context, CXCR4 mutation testing was not performed due to institutional limitations. While CXCR4 mutations may influence disease behavior and treatment response, they are not essential for diagnostic confirmation in WM.

Our patient presented with symptoms and imaging initially suggestive of a systemic lymphoproliferative disorder, which was confirmed as WM with an associated significant mesenteric mass. Standard first-line chemoimmunotherapy with bendamustine and rituximab (BR) was initiated, a regimen known for its efficacy in WM [13]. However, similar to the challenges that can arise in localized complications, the persistence and progression of obstructive symptoms despite two cycles of treatment underscore the limitations of systemic therapy alone in rapidly alleviating a significant, established mechanical problem. This necessitated surgical intervention, which, while not curative for the underlying WM, was lifesaving in the context of impending complete bowel obstruction and significantly improved the patient's quality of life.

One of the most compelling aspects of this case lies in the histopathological analysis of the resected specimen, which revealed extensive transmural intestinal ischemia and fibrosis, with no residual lymphomatous infiltration. These findings, observed after only two cycles of R-Benda, suggest an effective early cytoreductive response to treatment. Nonetheless, the persistence of a symptomatic mass indicates that irreversible structural damage had already occurred. It is likely that the initial tumor bulk, desmoplastic stromal reaction, and vascular compromise contributed to sustained ischemia and fibrosis, ultimately leading to mechanical obstruction. This supports the hypothesis that the clinical presentation was driven not only by viable tumour tissue, but also by secondary tissue changes resulting from prior infiltration and treatment effects.

Although an IgM flare is a recognized complication of rituximab-based therapy in WM and may contribute to transient symptom exacerbation, it is unlikely to have played a primary role in this case. The patient's symptoms and imaging abnormalities were present before treatment initiation, and the absence of lymphomatous infiltration in the surgical specimen, along with the presence of established fibrotic and ischemic changes, supports a chronic mechanical process rather than an acute biochemical flare.

To conclude, this case highlights the clinical importance of recognising rare but severe abdominal complications in WM that may not respond promptly to systemic therapy. While the pathological features are informative, the management decisions, particularly the timely shift to surgical intervention, represent the most clinically relevant contribution. To our knowledge, reports of WM requiring bowel resection due to subocclusive symptoms remain extremely limited. We believe this case adds valuable perspective on the role of surgery as a complementary strategy in the multidisciplinary management of select patients with WM presenting with severe abdominal involvement.

## Conclusions

Waldenström macroglobulinemia is a rare lymphoproliferative disorder that can occasionally present with atypical extranodal involvement, including the gastrointestinal tract. When patients develop abdominal symptoms, such as subocclusion, distension, or a palpable mass, clinicians should maintain a high index of suspicion for intestinal infiltration. In such scenarios, prompt surgical evaluation may be necessary, particularly when symptoms persist despite medical therapy. This case illustrates how an early multidisciplinary discussion, integrating hematology, surgery, radiology, and pathology, was essential for timely intervention and tailored management. The surgical resection not only provided symptomatic relief but also revealed critical histopathological findings that shaped the ongoing therapeutic plan. Timely decision-making in complex WM presentations may prevent serious complications and ultimately improve patient outcomes.
